# Cardiovascular Risk Factors among Individuals Incarcerated in an Arizona County Jail

**DOI:** 10.3390/ijerph18137007

**Published:** 2021-06-30

**Authors:** Ricky Camplain, Monica R. Lininger, Julie A. Baldwin, Robert T. Trotter

**Affiliations:** 1 Center for Health Equity Research, Northern Arizona University, Flagstaff, AZ 86011, USA; Julie.Baldwin@nau.edu (J.A.B.); Robert.Trotter@nau.edu (R.T.T.II); 2 Department of Health Sciences, Northern Arizona University, Flagstaff, AZ 86011, USA; 3 Department of Physical Therapy and Athletic Training, Northern Arizona University, Flagstaff, AZ 86011, USA; Monica.Lininger@nau.edu; 4 Department of Anthropology, Northern Arizona University, Flagstaff, AZ 86011, USA

**Keywords:** cardiovascular disease, hypertension, diabetes, smoking, binge drinking, correctional health, jail, NHANES

## Abstract

We aimed to estimate the prevalence of cardiovascular risk factors, including hypertension, diabetes, high cholesterol, cigarette smoking, alcohol consumption, and obesity among a sample of individuals incarcerated in an Arizona county jail and compare prevalence estimates to a matched non-institutionalized population. From 2017–2018, individuals housed at a county jail completed a cross-sectional health survey. We estimated the prevalence of hypertension, diabetes, cholesterol, overweight/obesity, cigarette smoking, binge drinking, and self-reported health among individuals incarcerated. We compared prevalence estimates of cardiovascular risk factors to a matched sample of 2017–2018 NHANES participants. Overall, 35.9%, 7.7%, and 17.8% of individuals incarcerated in jail self-reported hypertension, diabetes, and high cholesterol, respectively. Of individuals incarcerated, 59.6% were overweight or obese and 36.8% self-reported fair or poor general health. Over half of individuals incarcerated reported ever smoking cigarettes (72.3%) and binge drinking (60.7%). Compared to a matched sample of NHANES participants, individuals incarcerated in jail had a statistically higher prevalence of cigarette smoking and binge drinking. Screening of cardiovascular risk factors and providing preventive measures and interventions, such as healthy eating, physical activity, or pharmacological adherence interventions, while individuals are incarcerated may contribute to the prevention and management of cardiovascular risk factors and, eventually, cardiovascular disease.

## 1. Introduction

An estimated 12% of American adults have been diagnosed with cardiovascular disease and by 2030, 44% of the US population is projected to have some form of cardiovascular disease with direct medical costs expected to reach $818 billion [1]. While cardiovascular disease affects the total US population, inequities exist in the prevalence of cardiovascular disease; specifically among racial/ethnic minorities [2,3] and individuals of low socioeconomic status [4]. As 60% of incarcerated populations are ethnic and belong to racial minorities [5] and are disproportionately of low education and low income backgrounds [6], they may be at higher risk for cardiovascular disease compared to non-institutionalized populations [7,8]. Contributing to risk of cardiovascular disease may be underlying risk factors for cardiovascular disease, including hypertension, type II diabetes (diabetes), high cholesterol, overweight and obesity, cigarette smoking, and alcohol consumption that also disproportionately affect racial/ethnic minorities and individuals of a low socioeconomic status [9,10,11,12].

Although more than 20 million (9%) adults are currently or have been previously incarcerated along with the 12 million individuals that cycle through jails (facilities that typically house individuals awaiting adjudication or serving sentences < 1 year) each year [13], limited research has been conducted to assess cardiovascular risk factors among individuals incarcerated. What little research there is, cardiovascular risk factors may be substantial among individuals incarcerated [8,14,15,16,17,18]. Despite the potential burden of cardiovascular disease risk factors among incarcerated individuals, most research to date is among individuals incarcerated in prison, long-term facilities housing individuals serving sentences ≥ 1 year. Prisons are distinctively different from jails, as jails are short-term facilities designed to hold individuals awaiting trial or serving short sentences. Most of the 20 million Americans [13] that were previously or are currently incarcerated were or are exclusively in jails and 95% of releases from correctional facilities are from jails [19]. Excluding jail populations in research ignores a substantial proportion of the incarcerated populations; thus, potentially underestimating the true burden of cardiovascular risk factors among individuals incarcerated. Thus, the objective of the study was to estimate the prevalence of cardiovascular risk factors, including hypertension, diabetes, high cholesterol, smoking, alcohol consumption, and obesity among a sample of individuals incarcerated in an Arizona county jail. We additionally aimed to compare prevalence of cardiovascular risk factors among individuals incarcerated in jail to non-institutionalized adults using the National Health and Nutrition Examination Survey (NHANES).

## 2. Materials and Methods

### 2.1. Study Population

From 2017–2018, individuals housed at the Coconino County Detention Facility (CCDF) in Flagstaff, Arizona were recruited to participate in a cross-sectional health survey [20]. CCDF houses, on average, 450 men and women daily, and consists of 21 housing units, which are segmented by sex (male and female), internal risk assessment (low, medium, and high security), and known conflicts among individuals housed in the facility. Most individuals incarcerated at CCDF are between the ages of 18 and 34 years, male, and white, American Indian/Alaska Native (AI/AN), or Latino/Latina [21]. Participants were recruited from housing units based on a non-random stratified sampling strategy. Individuals were eligible if they were ≥18 years and able to read English. Individuals housed in juvenile, administrative confinement, severe mental illness, and administration dorms were excluded. Of the 204 participants who completed the survey, five piloted the questionnaire for a final sample of 199. The study was approved by Northern Arizona University Institutional Review Board and participants gave written, informed consent.

### 2.2. Demographics

Demographic information was self-reported. Age during the interview was determined from date of birth. Sex was categorized as male or female. Race/ethnicity was categorized as Hispanic/Latino, non-Hispanic White, non-Hispanic Black, or Other Race. Most of our sample were AI/AN however, to be comparable to NHANES, they were grouped into an “other” category. Education was categorized as less than a high school diploma or General Educational Development (GED), a high school diploma or GED, or some college or greater. Income was reported in US dollars and categorized as $0–9999, $10,000–19,999, ≥$20,000, and “Don’t know”. Health insurance coverage was categorized as Medicare or Medicaid, other (employer, private insurance, CHIP, military health care, self-insured, Indian Health Services), or no coverage.

### 2.3. Cardiovascular Disease Risk Factors

All cardiovascular risk factors, including hypertension, diabetes, cholesterol, overweight and obesity, cigarette smoking, alcohol use, and general health were self-reported (Table 1). Body mass index (BMI) was calculated as weight in kilograms divided by height in meters, squared and categorized as normal (<25), overweight (25–29), and obese (≥30). Binge drinking was categorized as males having five or more drinks of alcohol on one occasion or females having four or more drinks of alcohol on at least one occasion. Self-reported general health was categorized as excellent, very good, good, fair, or poor.

### 2.4. Cardiovascular Disease

Participants self-reported whether a doctor or other health professional told them they had heart failure, coronary heart disease, angina, a myocardial infarction, or a stroke.

### 2.5. NHANES

NHANES is a cross-sectional survey designed to monitor the health and nutritional status of the civilian, non-institutionalized US population using highly stratified, multistage probability design [22]. NHANES consists of interviews conducted in participants’ homes and standardized health examinations conducted in mobile examination centers. Data from NHANES 2017–2018 were used for these analyses.

For demographics, age, sex (male/female), race/ethnicity (Mexican American, Other Hispanic, Non-Hispanic White, Non-Hispanic Black, Other Race—Including Multi-Racial), education, annual household income, and health insurance (Medicare, Medicaid, other coverage, no coverage) were used. To note, the “Other Race” category cannot be further disaggregated.

#### 2.5.1. Cardiovascular Disease Risk Factors

All cardiovascular risk factors, including hypertension, diabetes, cholesterol, overweight and obesity, cigarette smoking, alcohol use, and general health were self-reported (Table 1).

#### 2.5.2. Cardiovascular Disease

NHANES participants self-reported whether a doctor or other health professional told them they had heart failure, coronary heart disease, angina, a myocardial infarction, or a stroke.

### 2.6. Data Matching

Participants from CCDF were matched to a random sample of NHANES participants on age at interview, sex, education, and type of health insurance coverage using the PROC SURVEYSELECT procedure by selecting samples within the independent, stratified groups. Participants were not matched on race/ethnicity as important racial/ethnic groups in NHANES are limited to an “Other Race” category that cannot be further disaggregated.

### 2.7. Statistical Analysis

Demographic characteristics and cardiovascular risk factors were presented as frequencies and relative frequencies by population (CCDF and NHANES participants). Odds ratios (ORs) and 95% confidence intervals (CI) were estimated using logistic regression to determine the differences in cardiovascular risk factors between CCDF and NHANES participants. All models were adjusted for annual household income. Analyses were conducted using SAS version 9.4 (SAS Institute Inc., Cary, NC, USA).

## 3. Results

Among the 197 participants incarcerated at CCDF and the 196 matched NHANES participants, most were between the ages of 18 and 44 years, male, had a high school diploma or more, and had health insurance coverage at the time of incarceration (Table 2). Most participants incarcerated at CCDF were a race/ethnicity other than Hispanic/Latino, non-Hispanic White, or non-Hispanic black (59.5%). From previous work, most individuals incarcerated at CCDF are AI/AN [21]. However, because NHANES categorizes AI/AN participants as “other” and are not able to be identified, we created a comparable category. Most participants incarcerated had an annual household income of less than $10,000 (45.3%) while selected NHANES participants had an annual household income of ≥$20,000 (77.3%). Finally, a small proportion of participants from CCDF and NHANES reported that a physician or health professional had told them they had cardiovascular disease.

### 3.1. Hypertension

Compared to NHANES participants (27.1%), a higher proportion of participants from CCDF reported being told by a healthcare professional they had hypertension (35.9%, Table 3). Results were similar for being told they had hypertension by a healthcare professional on two separate occasions. In contrast, compared to NHANES participants (23.5%), a lower proportion of participants from CCDF reported ever being prescribed medication for hypertension (19.8%) with similar findings regarding taking a prescription for hypertension at the time of the survey. Despite the different patterns of hypertension-related outcomes, no associations between incarceration status (individuals incarcerated at CCDF vs. NHANES participants) and hypertension-related outcomes were statistically significant (Table 4).

### 3.2. Diabetes

Compared to NHANES participants (6.1%), a higher proportion of participants from CCDF reported being told by a healthcare professional they had diabetes (7.7%, Table 3). Similarly, a higher proportion of participants from CCDF reported taking insulin or a diabetic pill at the time of the survey. However, no associations between incarceration status and diabetes-related outcomes were statistically significant (Table 4).

### 3.3. High Cholesterol

Compared to NHANES participants (20.9%), a lower proportion of participants from CCDF reported being told by a healthcare professional they had high cholesterol (17.8%, Table 3). A lower proportion of participants from CCDF reported ever being prescribed medication for high cholesterol (9.1%) compared to NHANES participants (15.3%). However, no associations between incarceration status and high cholesterol-related outcomes were statistically significant (Table 4).

### 3.4. Overweight and Obesity

Compared to NHANES participants (27.1%), a higher proportion of participants from CCDF had a normal BMI (40.4%) while a lower proportion of participants from CCDF were in the obese category (22.8%) compared to NHANES participants (37.8%, Table 3). However, the association between incarceration status and BMI was not statistically significant (Table 4).

### 3.5. Cigarette Smoking

Compared to NHANES participants (49.0%), a higher proportion of participants from CCDF reported ever smoking cigarettes (72.3%, Table 3). Compared to NHANES participants, participants form CCDF had higher odds of ever cigarette smoking (OR = 2.58, 95% CI: 1.56, 4.29, Table 4).

### 3.6. Alcohol

Compared to NHANES participants (35.5%), a higher proportion of participants from CCDF reported binge drinking in the 30 days prior to being incarcerated (60.7%, Table 3). Compared to NHANES participants, participants form CCDF had higher odds of binge drinking (OR = 2.63, 95% CI: 1.49, 4.66, Table 4).

### 3.7. General Health

Compared to NHANES participants (72.4%), a lower proportion of participants from CCDF reported good, very good, or excellent general health (63.2%). However, the association between incarceration status and general health was not statistically significant (Table 3).

## 4. Discussion

Each year, 9 million Americans cycle in and out of the 3200 jails in the US [23] and cardiovascular disease is substantial among individuals incarcerated in jail and prison [7], which may be due to the high prevalence of cardiovascular risk factors among individuals incarcerated in jail. We found that although a higher proportion of individuals incarcerated in a rural Arizona county jail reported hypertension, diabetes, cigarette smoking, binge drinking, and fair or poor general health compared to a matched non-institutionalized sample, incarceration status was only associated with cigarette smoking and binge drinking health behaviors. Our study is the first to estimate the differences between a southwestern US incarcerated population and a matched non-institutionalized population and the first, overall, to match with NHANES participants, a nationally representative sample of the US population.

A higher proportion of individuals incarcerated reported having hypertension compared to NHANES participants. Our findings are similar to previous research indicating those who were previously incarcerated had increased risk of hypertension compared to individuals who were never incarcerated [8,15,18] and individuals in prison have higher prevalence or odds of hypertension compared to the general population [16]. Although more incarcerated participants indicated having hypertension, a higher proportion of NHANES participants indicated they were ever prescribed or were currently taking hypertensive medication. Although not statistically significantly different, there may be a few reasons for this discrepancy. The jail is required to provide appropriate medication to individuals incarcerated [24]. However, due to the lack of medical supplies, staff, and effective screening mechanisms, incarcerated individuals may not receive prescribed medications [25]. Regarding a lower proportion of participants incarcerated never being prescribed hypertensive medication compared to NHANES counterparts, there is little information regarding prescribing behaviors in hypertension medication by physicians. In what little research has been done, there may be implicit racial bias in prescribing practices as racial/ethnic minorities are prescribed analgesics at a lower rate compared to white participants. However, there is no data on hypertensive medications. Without access to appropriate hypertensive treatments, individuals may be at a higher risk for developing cardiovascular disease.

We found among our relatively young (78% under the age of 45) jail population that 7.7% indicated they had diabetes. Our estimates were slightly higher compared to 5.1% of individuals incarcerated in prison [13]. We may have a higher proportion as most of the individuals incarcerated at CCDF are AI/AN. Prevalence of diagnosed diabetes is highest among AI/AN populations compared to other race/ethnicities [26]. Additionally, early onset diabetes impacts AI/AN populations more often than for other racial/ethnic groups [27]. However, we found that individuals incarcerated in jail had a similar prevalence of diabetes compared to NHANES participants. This is contradictory to previous research in which individuals incarcerated in jail and prison had a higher prevalence of diabetes compared to non-institutionalized adults [8]. A major limitation of the current study that may have impacted the findings with regard to the association between incarceration status with diabetes and other cardiovascular risk factors, was the fact that we were unable to match our sample on race/ethnicity. NHANES groups AI/AN participants, in addition to other racial/ethnic groups and those who may identify as mixed race, into an “other” category. Without the ability to disaggregate “other” race/ethnicities for NHANES participants, race/ethnicity may have implications for our findings. Specifically, race/ethnicity may be a critical confounder or effect measure modifier that may impact findings of associations by race/ethnicity. Future research should include incarcerated and non-institutionalized AI/AN and adults of other race/ethnicities to be able to determine if there are differences in incarceration and cardiovascular risk factors by race/ethnicity.

The reported prevalence of high cholesterol was lower in individuals incarcerated in jail compared to NHANES participants. Additionally, previous research has found that 38% of prisoners have elevated mean blood cholesterol levels [18], higher than the 17% reported by our jail study participants. A limitation was that we could not verify cholesterol levels of participants in the jail, as this was self-reported and based upon whether a health care professional had told them they had high cholesterol. Due to a change in diet and exercise, cholesterol levels may increase during incarceration. Individuals in prison with longer lengths of stays compared to those in jail, may have longer exposure to changes in diet and exercise and produce a higher prevalence of high cholesterol.

Although a smaller proportion of individuals incarcerated in jail were overweight or obese compared to NHANES participants, we found that more than half of individuals incarcerated in jail were overweight or obese, similar to previous reports of 50–68% [8]. With greater variability in length of stay among those in jail compared to prison, individuals in prison were more likely to be obese than those in jail, with 61–77% being overweight or obese [8]. Cigarette smoking, found to be significantly higher among individuals incarcerated, is associated with a lower BMI [28], which may partially explain our findings.

Over 70% of our participants were ever smokers, which is higher among individuals incarcerated in jail compared to NHANES participants (49.0%). Smoking is the second most common cardiovascular disease risk factor among individuals incarcerated with a smoking prevalence that is up to three times that of the general population [14]. Over 60% of individuals with a prior incarceration were current or former tobacco users compared to 31% of those who were never incarcerated [15]. Binge drinking also increases risk of cardiovascular disease [29]. We found that 60% of individuals incarcerated in jail reported binge drinking behavior. This was higher compared to a previous study that found that 25–48% of individuals in prison or jail reported binge drinking [30]. Additionally, binge drinking was higher among individuals incarcerated in jail compared to NHANES participants. Although we did not see statistically significant differences in other risk factors, the stark differences in health behavior risk factors for cardiovascular disease were clear.

As our study is cross sectional in nature, we cannot determine temporality of incarceration and cardiovascular risk factors. There are mechanistically sound reasons that explain the high proportion of incarcerated individuals that have cardiovascular risk factors, specifically behavior-related risk factors. One potential mechanism by which incarceration may be associated with a high presence of cardiovascular risk factors may be that social groups with high cardiovascular disease risk are over-represented in the incarcerated population; specifically, those with lower socioeconomic status or racial/ethnic minorities [21]. Previous research has explored this mechanism and found that incarcerated individuals may have elevated cardiovascular risk, even when adjusted for socioeconomic factors [8]. Additionally, incarceration, along with other social factors associated with incarceration, may cause high levels of stress, which may affect coping behaviors such as smoking and alcohol use and play a direct role in the pathophysiology of cardiovascular disease through the sympathetic and parasympathetic systems that may include the increase of cardiovascular risk factors [31].

To note, cardiovascular risk factors were self-reported. Previous research has found that self-reports of health conditions may underestimate the true prevalence, possibly driven by individuals who are undiagnosed [32]. Height has been shown to be systematically overreported and weight underreported [33]. In addition, underestimating alcohol consumption increases with heavy consumption [34]. Thus, our estimates of health conditions and binge drinking may underestimate the true burden among individuals incarcerated in jail. Despite variations in validity and accuracy of self-reported cardiovascular risk factors, we were able to directly compare results among incarcerated individuals with prevalence estimates among a matched sample of NHANES participants as the questions given to participants incarcerated in jail were identical to NHANES.

Currently, there are no population-level studies of cardiovascular risk factors among individuals in jail and most national household-based surveys do not include questions on recent incarceration or include individuals incarcerated in jail. The ability to match our study population in a rural Arizona County with a non-institutionalized population (the first to match with NHANES) allows a comparison of cardiovascular risk factors by incarceration status. Specifically, we were able to ask participants at CCDF identical questions to NHANES participants. Our findings indicate that a high proportion of individuals incarcerated in jail may have risk factors for cardiovascular disease and thus, increasing risk for adverse events during incarceration and after release. Cardiovascular disease risk factor screening upon admission is common among state prison systems [35]. However, because most people in the criminal justice system go through jails and are released more frequently to the community, jail may be an appropriate place for screening cardiovascular risk factors, specifically behaviors such as alcohol consumption and cigarette smoking, and education on how to manage these risk factors. Short-term smoking cessation programs are comparable to traditional smoking cessation interventions and may be adapted for individuals incarcerated in jails [36]. This may not only reduce the burden on the correctional health care system but also the community health care systems.

How the jail environment affects prevention and self-management strategies for diet, physical activity, pharmacological adherence behaviors, and other strategies among individuals currently incarcerated is understudied [7]. The availability of low-fat or low-sodium diets [37], ability to exercise [38], and management of their own medications are all largely out of the control of most individuals incarcerated in jail and may contribute to the development of cardiovascular risk factors or gaps in continuity of care. If an individual is diagnosed with a cardiovascular disease risk factor in an incarceration setting, understanding how to care for oneself may prove difficult as they are not allowed to manage their own medications and have limited freedom to exercise and make healthy food choices. In addition to how the jail environment affects prevention and self-management of typical cardiovascular risk factors, the jail environment itself may impact cardiovascular risk though other mechanisms that were not considered in this study such as stress [39], anxiety, depression, other mental health disorders [40], as well as indoor air pollution [41].

## 5. Conclusions

Individuals incarcerated in jail have a higher prevalence of self-reported cigarette smoking and binge drinking compared to a matched NHANES sample, which may lead to increased risk of cardiovascular disease among this population. Screening and providing preventive measures and interventions, such as healthy eating, physical activity, or pharmacological adherence interventions, while individuals are incarcerated may contribute to the prevention and management of cardiovascular risk factors and, eventually, cardiovascular disease.

## Figures and Tables

**Table 1:** Cardiovascular disease risk factor measurements among Coconino County Detention Facility (CCDF) participants and matched National Health and Nutrition Examination Survey (NHANES) participants, 2017–2018.

Cardiovascular Disease Risk Factor Measure	Measure Definition	
	CCDF	NHANES
Hypertension	“Have you ever been told by a doctor or other health professional that you had hypertension, also called high blood pressure? [If high blood pressure only during pregnancy, select no.]”	“Have you ever been told by a doctor or other health professional that had hypertension, also called high blood pressure?”
Two diagnoses of hypertension	“Were you told on 2 or more different visits that you had hypertension, also called high blood pressure?”	“Were you told on 2 or more different visits that you had hypertension, also called high blood pressure?”
Hypertension prescription, ever	“Because of your high blood pressure/hypertension, have you ever been told to take prescribed medicine?”	Because of your (high blood pressure/hypertension), have you ever been told to… take prescribed medicine?”
Hypertension prescription, current	“Are you now taking a prescribed medicine?”	“Are you now taking prescribed medicine?”
Diabetes	“[Other than during pregnancy], Have you ever been told by a doctor or other health professional that you have diabetes or sugar diabetes?”	“[Other than during pregnancy], Have you ever been told by a doctor or other health professional that have diabetes or sugar diabetes?”
Taking insulin at time of survey	“Are you now taking insulin?”	“Are you now taking insulin?”
Taking a diabetic pill at time of survey	“Are you now taking diabetic pills to lower your blood sugar? These are sometimes called oral agents or oral hypoglycemic agents.”	“Are you now taking diabetic pills to lower your blood sugar? These are sometimes called oral agents or oral hypoglycemic agents.”
High cholesterol	“Have you ever been told by a doctor or other health professional that your blood cholesterol level was high?”	“Have you ever been told by a doctor or other health professional that your blood cholesterol level was high?”
High cholesterol prescription, ever	“To lower your blood cholesterol, have you ever been told by a doctor or other health professional to take prescribed medicine?”	“To lower your blood cholesterol, have you ever been told by a doctor or other health professional… to take prescribed medicine?”
Body Mass Index	“How tall are you without shoes?—Enter number of feet.” “How tall are you without shoes?—Enter number of inches.” “How much do you weigh without clothes or shoes?—Enter weight in pounds.”	“How tall are you without shoes?”—Enter height in feet and inches or meters and centimeters. “How much do you weigh without clothes or shoes?”—Enter eight in pounds or kilograms.
Ever cigarette smoker	“Have you smoked at least 100 cigarettes in your entire life?”	“Have you smoked at least 100 cigarettes in your entire life?”
Binge Drinking	“During the 30 days before admission, on how many days did you have X or more drinks of alcohol in a row, that is, within a couple of hours?” X = 5 for men and X = 4 for women	“Considering all types of alcoholic beverages, during the past 30 days, how many times did you have X or more drinks on an occasion?” X = 5 for men and X = 4 for women
General Health	“Would you say your health in general is…”	“Would you say your health in general is…”

Note: NHANES 2017–2018 Questionnaire Data information is available here: https://wwwn.cdc.gov/nchs/nhanes/Search/DataPage.aspx?Component=Questionnaire&CycleBeginYear=2017 (accessed on: 16 April 2021).

**Table 2:** Characteristics of individuals incarcerated in Coconino County Detention Facility and matched NHANES participants, 2017–2018.

Characteristic	CCDF (*n* = 197)		NHANES (*n* = 196)	
	*n*	%	*n*	%
Age at interview (years)				
18–24	23	11.7	23	11.7
25–34	71	36.0	71	36.2
35–44	60	30.5	59	30.1
45–54	31	15.7	31	15.8
≥55	12	6.1	12	6.1
Sex				
Female	42	21.3	42	21.4
Male	155	78.7	154	78.6
Race/Ethnicity				
Hispanic/Latino	29	14.7	51	26.0
Non-Hispanic White	48	24.4	56	28.6
Non-Hispanic Black	2	1.0	51	26.0
Other ^a^	118	59.9	38	19.4
Education				
Less than a high school diploma or GED	60	30.5	60	30.6
High school diploma or GED	76	38.5	76	38.8
Some college or greater	61	31.0	60	30.6
Annual household income				
$0–9999	87	45.3	10	5.1
$10,000–19,999	19	9.9	23	11.7
≥$20,000	53	27.6	136	69.4
Don’t know ^b^	33	17.2	27	13.8
Health insurance coverage				
Yes	154	78.6	152	78.4
No	42	21.4	42	21.6
Missing	1		2	
Type of health insurance coverage				
Medicare	10	5.1	9	4.6
Medicaid	105	53.9	105	54.1
Other coverage ^c^	38	19.5	38	19.6
No coverage	42	21.5	42	21.7
Missing	2		2	
Prevalent cardiovascular disease ^d^				
Yes	8	4.1	10	5.1
No	189	95.9	186	94.9

Abbreviations: National Health and Nutrition Examination Survey (NHANES). ^a^ Other included participants who identified as American Indian/Alaska Native, Asian or Pacific Islander, or other. ^b^ Refused to answer or did not know. ^c^ Other coverage included employer, private insurance, Children’s Health Insurance Program, military health care, self-insured, Indian Health Services. ^d^ Heart failure, coronary heart disease, angina, myocardial infarction, or stroke.

**Table 3:** Prevalence of cardiovascular disease risk factors among individuals incarcerated in Coconino County Detention Facility and NHANES participants, 2017–2018.

Cardiovascular Disease Risk Factor	CCDF (*n* = 197)		NHANES (*n* = 196)	
	*n*	%	*n*	%
Health Conditions				
Hypertension	69	35.9	53	27.1
Two diagnoses of hypertension	52	26.4	45	23.0
Hypertension prescription, ever	39	19.8	46	23.5
Hypertension prescription, current	23	11.7	32	16.3
Diabetes	15	7.7	12	6.1
Taking insulin at time of survey	7	3.6	4	2.0
Taking a diabetic pill at time of survey	14	7.1	9	4.6
High cholesterol	34	17.8	41	20.9
High cholesterol prescription, ever	18	9.1	30	15.3
Body Mass Index				
Normal (<25)	78	40.4	51	27.1
Overweight (25–29)	71	36.8	66	35.1
Obese (≥30)	44	22.8	71	37.8
Missing	4		8	
**Health Behaviors**				
Ever cigarette smoker	141	72.3	96	49.0
Binge Drinking ^a^	119	60.7	44	35.5
**General Health**				
Excellent	16	8.3	20	11.5
Very Good	39	20.2	35	20.1
Good	67	34.7	71	40.8
Fair	56	29.0	44	25.3
Poor	15	7.8	4	2.3
Missing	4		22	

Abbreviations: National Health and Nutrition Examination Survey (NHANES) ^a^ During the 30 days before admission to jail, how many times did you have X or more drinks in a day? (X = 5 for men and 4 for women), and a response of 1 or more is considered positive for unhealthy alcohol use. A drink is defined as 12 ounces of beer, 5 ounces of wine, or 1.5 ounces of 80 proof spirits.

**Table 4:** Association between incarceration status (individuals incarcerated at Coconino County Detention Facility compared to NHANES participants) and cardiovascular disease risk factors, 2017–2018 (N = 393).

Cardiovascular Disease Risk Factor	OR	95% CI	
Health Conditions			
Hypertension			
NHANES	1.00		
Incarcerated	1.37	0.81	2.33
Two diagnoses of hypertension			
NHANES	1.00		
Incarcerated	1.06	0.61	1.87
Hypertension prescription, ever			
NHANES	1.00		
Incarcerated	0.66	0.36	1.21
Hypertension prescription, current	1.00		
NHANES	0.56	0.27	1.14
Incarcerated			
Diabetes			
NHANES	1.00		
Incarcerated	1.78	0.69	4.58
Taking a diabetic pill at time of survey			
NHANES	1.00		
Incarcerated	2.49	0.92	6.74
High Cholesterol			
NHANES	1.00		
Incarcerated	1.03	0.56	1.88
High cholesterol prescription, ever			
NHANES	1.00		
Incarcerated	0.69	0.33	1.44
Body Mass Index ^a^			
NHANES	1.00		
Incarcerated	0.74	0.45	1.25
**Health Behaviors**			
Ever Cigarette Smoker			
NHANES	1.00		
Incarcerated	2.58	1.56	4.29
Binge Drinking			
NHANES	1.00		
Incarcerated	2.63	1.49	4.66
**General Health**			
General Health ^b^			
NHANES	1.00		
Incarcerated	0.98	0.57	1.71

Abbreviations: National Health and Nutrition Examination Survey (NHANES); Odds ratio (OR); Confidence interval (CI). All models were adjusted for annual household income. ^a^ Normal vs. overweight and obese. ^b^ Excellent, very good, and good vs. fair and poor. To note, models for “taking insulin at time of survey” did not converge and therefore are not presented in Table 3.

## Data Availability

Appropriately deidentified data presented in this study are potentially available on approved request from the corresponding author. The data are not publicly available due to ethical considerations for the population involved and would require approval from the County Criminal Justice Coordinating Council and the NAU Data Safety Committee.
